# The meta and bioinformatics analysis of fascin expression in gastric cancer: a potential marker for aggressiveness and worse prognosis

**DOI:** 10.18632/oncotarget.22325

**Published:** 2017-11-06

**Authors:** Hua-Chuan Zheng, Shuang Zhao

**Affiliations:** ^1^ Department of Experimental Oncology and Animal Center, Shengjing Hospital of China Medical University, Shenyang 110004, China

**Keywords:** fascin, gastric cancer, meta analysis, bioinformatics analysis

## Abstract

Fascin is a FSCN1-encoded actin bundling protein, and positively associated with proliferation, migration and metastasis of malignancies. Here, we performed a systematic meta and bioinformatics analysis through multiple online databases up to March 14, 2017. We found up-regulated fascin expression in gastric cancer, compared with normal mucosa (p<0.05). Fascin expression was positively with lymph node metastasis, TNM staging and worse prognosis of gastric cancer (p<0.05). According to bioinformatics database, *FSCN1* mRNA expression was higher in gastric cancer than normal tissues (p<0.05). According to Kaplan-Meier plotter, we found that a higher *FSCN1* expression was negatively correlated with overall and progression-free survival rates of all cancer patients, even stratified by aggressive parameters (p<0.05). These findings indicated that fascin expression might be employed as a potential marker to indicate gastric carcinogenesis and subsequent progression, even prognosis.

## INTRODUCTION

Fascin is a 55 kDa monomeric actin filament bundling protein originally isolated from sea urchin egg, and contributes to increased proliferation, altered β1 integrin distribution, enhanced invasive capacity and dedifferentiation status [1, 2]. Fascin can directly interact with the microtubule cytoskeleton, and control fascin-dependent focal adhesion dynamics and cell migration speed, which is due to fascin-FAK-src complex formation [3]. However, E3 ligase Smurf1 monoubiquitinates fascin at Lys247 and Lys250, which decreases the fascin bundling EC50, delays the initiation of bundle assembly, and accelerates the disassembly of existing bundles [4].

Fascin protein is critical for TGFβ -induced invasion and filopodia formation in spindle- shaped tumor cells through the canonical Smad-dependent pathway [5, 6]. GATA3 abrogates Smad4 -mediated fascin overexpression, invadopodium formation, and invasion of breast cancer cells by abolishing the interaction between Smad4 and its DNA binding elements [7]. Snyder et al. [8] found that Stat3-NF-κB complex was necessary for fascin expression in metastatic breast cancer cells in response to IL-6 and TNF-α. The prometastatic RSK2-CREB pathway increased fascin expression to promote tumor metastasis [9, 10]. Osanai et al. [11] demonstrated that CYP26A1 up-regulated fascin, and subsequently enhanced cell apoptotic resistance, anchorage-independent growth, mobility, invasion and escaped premature senescence in breast cancer cells.

Conditional expression of fascin decreased mice survival and increased tumor burden compared to control animals, and fascin expression accelerated tumor progression and formation of invasive adenocarcinoma in adult tumor-bearing animals [12]. *FCSN1*- deficient KRas(G12D) p53(R172H) Pdx1-Cre mice had longer survival times, delayed onset of pancreatic ductal adenocarcinoma (PDAC), and a lower PDAC tumor burdens than KPC mice [13]. Fascin is widely expressed in the mature dendritic cells, mesencymal cells, endothelial cells and neurons of the human, but low or absent in adult epithelia. Recent data have highlighted that fascin is up-regulated in many human cancer, and correlated with the clinical aggressiveness and poor patient survival [14, 15]. In the present study, we performed both meta- and bioinformatics analyses to clarify clinicopathological and prognostic significances of fascin expression in gastric carcinogenesis and subsequent progression.

## RESULTS

### Characteristics of eligible studies

**Figure 1:**
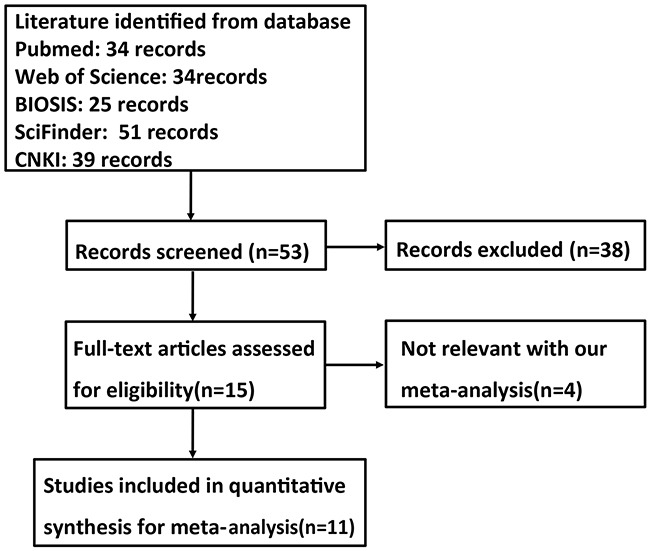
Flow diagram of the selection process in this meta-analysis

As shown in Figure 1 and Table 1, a total of 11 articles on the relationship between fascin expression and cancer risk, clinicopathological or prognostic parameters of gastric cancer were retrieved for our meta-analysis by immunohistochemistry in PubMed, Web of Science, BIOSIS, SciFinder and CNKI. Only 7 articles contained the samples of normal gastric mucosa [16–22]. There appeared the comparison between fascin expression and clinicopathological characteristics of gastric cancer in 11 studies, including sex, depth of invasion, lymph node metastasis, TNM staging and Lauren's classification [16–26].

**Table 1:** Main characteristics of eligible studies

First author	Year	Country	Ethnicity	AS	Cases	Control	Risk to cancer	Outcome	Quality
Hashimoto Y	2003	Japan	Asian	Dako	214			Negative	8
Tsai WC	2007	China	Asian	Neomarker	100			Negative	8
Li XH	2008	Japan	Asian	Neomarker	509	138	Up	Negative	9
Kim SJ	2012	China	Asian	Dako	471			Negative	8
Tu L	2016	China	Asian	Cell Signaling	204	204	Up	Negative	9
Li K	2009	China	Asian	Dako	76	76	Up		8
Peng LT	2011	China	Asian	Neomarker	52	52	Up		8
Gan FL	2013	China	Asian	Neomarker	67	67	Up		8
Rao P	2013	China	Asian	Neomarker	150			Negative	8
Li M	2014	China	Asian	Boster	90	90	Up		8
Li S	2015	China	Asian	Maxin	107	40	Up		8

### Association between fascin expression and cancer susceptibility of gastric mucosa

**Figure 2:**
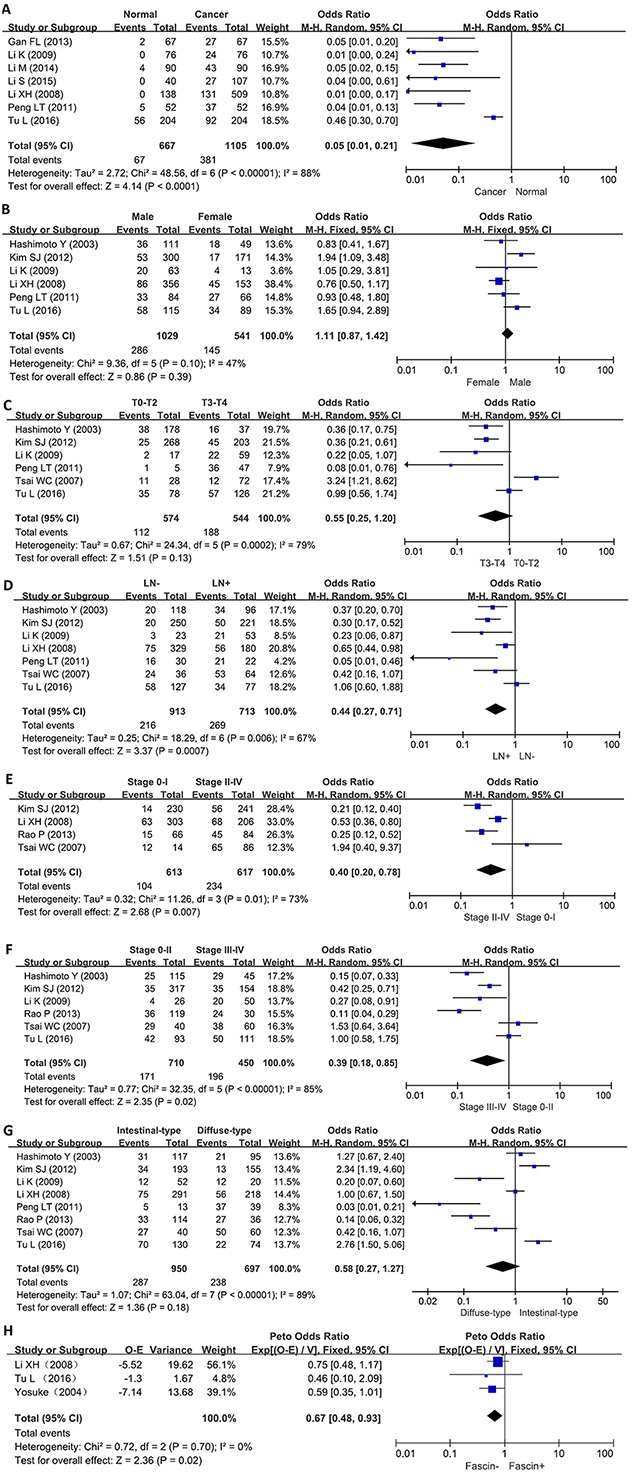
Forest plot for the relationship between fascin expression and clinicopatholoiocal parameters of gastric cancer **(A)** gastric carcinogenesis (cancer vs normal mucosa); **(B)** correlation between sex and fascin expression (male vs female); **(C)** correlation between depth of invasion and fascin expression (Tis-2 vs T3-4); **(D)** correlation between lymph node metastasis (LN) and fascin expression (LN- vs LN+); **(E)** correlation between TNM staging and fascin expression (0-I vs II-IV); **(F)** correlation between TNM staging and fascin expression (0-II vs III-IV); **(G)** correlation between differentiation and fascin (intestinal-type vs diffuse-type); **(H)** correlation between survival rate and fascin expression (fascin - vs fascin +).

We analyzed the association between fascin expression and cancer susceptibility of gastric normal mucosa in 7 studies with 1105 cancers and 667 controls. As a result, we found up-regulated fascin expression in gastric cancer, compared with normal mucosa (p<0.0001, Figure 2A).

### Association between fascin expression and clinicopathological parameters of gastric cancer

As shown in Figure 2B, there was no difference in fascin expression between male and female patients with gastric cancer (p>0.05). Fascin expression was not associated with T staging of gastric cancer (Figure 2C, p>0.05). A higher fascin expression was detected in gastric cancer with than with out lymph node involved (Figure 2D, p<0.0007). Fascin expression was positively linked to TNM staging regardless of subgrouping methods (Figures 2E and 2F, p<0.05). Intestinal-type carcinoma showed similar level of fascin expression to diffuse-type one (Figure 2G, p>0.05).

### Association between fascin expression and survival rate of gastric cancer

As indicated in Figure 2H, the pooled results from 3 studies demonstrated a significant association between fascin expression and overall survival in the patients with gastric cancer (HR = 0.67, 95% CI: 0.48-0.93, p=0.02). Results showed that fascin overexpression had an unfavorable prognostic value in gastric cancer patients.

### Publication bias

**Figure 3:**
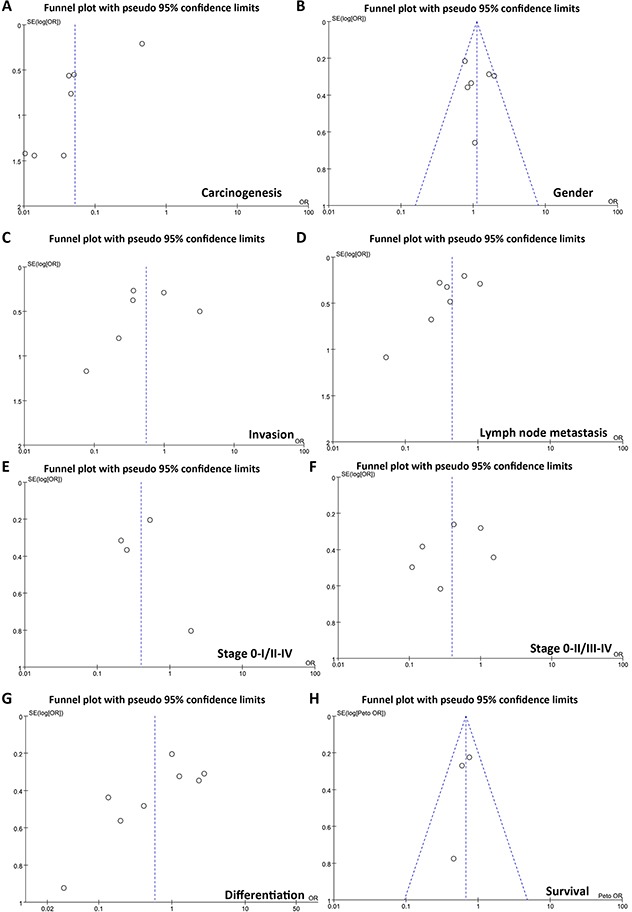
Funnel plot for publication bias test between fascin expression and gastric carcinogenesis or subsequent progression The bias was analyzed about risk degrees of fascin expression in gastric mucosa for gastric carcinogenesis **(A)**. Additionally, it was tested between fascin expression and clinicopathlogical features of gastric cancer, including age **(B)**, depth of invasion **(C)**, lymph node metastasis **(D)**, TNM staging **(E, F)**, and differentiation **(G)** and prognosis **(H)**.

The heterogeneity test was performed as shown in Figure 3. Sensitivity analysis was used to evaluate individual study's influence on the pooled results by deleting one single study each time from pooled analysis. As a result, T-staging result of fascin expression in Tsai's study had a significant effect on the pooled OR. When this study was excluded, the heterogeneity test was significantly reduced (data not shown).

### The clinicopathological and prognostic significance of *FSCN1* expression in gastric cancers

**Figure 4:**
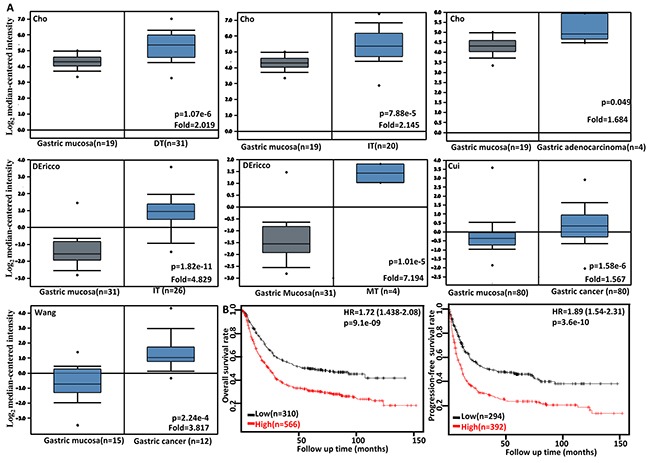
The clinicopathological significances of *FSCN1* mRNA expression in gastric cancer Cho's, DErrico's, Cui's and Wang's datasets were employed for bioinformatics analysis to analyze *FSCN1* mRNA expression during gastric carcinogenesis. A higher *FSCN1* expression was detectable in gastric cancer than that in normal gastric mucosa, even stratified into intestinal-(IT), diffuse-(DT), and mixed-type (MT) carcinomas by Lauren's classification (**A**, p<0.05). According to the data from KM plotter, *FSCN1* mRNA expression was positively related to both overall and progression-free survival rates of the patients with gastric cancer **(B)**. HR, hazard ratio.

Then, we used Cho's, DErrico's, Cui's and Wang's datasets to perform bioinformatics analysis and found that *FSCN1* expression was higher in gastric cancer than normal tissues, even stratified into intestinal-, diffuse- and mixed-type carcinoma (Figure 4A, p<0.05). According to Kaplan-Meier plotter, we found that a higher *FSCN1* expression was positively correlated with overall and progression-free survival rates of all cancer patients, even stratified by gender, TNM staging, lymph node involvement, any treatment (i.e. surgery alone, 5-FU-base adjuvant and other), Lauren's classification and Her2 expression (Figure 4B and Table 2, p<0.05). It was the same for the patients with M0, moderately- differentiated, perforation-negative, T2 or T3 cancer (Table 2, p<0.05). T4 or poorly- differentiated cancer patients with high *FSCN1* expression showed a low progression-free survival time than those with its low expression (p<0.05).

**Table 2:** The prognostic significance of *FSCN1* mRNA in gastric cancer

Clinicopathological features	Overall survival		Progression-free survival	
	Hazard ratio	p	Hazard ratio	p
Sex				
Female	2.06 (1.3 − 3.27)	0.0017	2.32 (1.41 − 3.8)	0.00063
Male	1.89 (1.5 − 2.38)	3e−08	2.02 (1.59 − 2.57)	6.3e−09
T				
2	2.32 (1.52 − 3.55)	6.3e−05	1.93 (1.27 − 2.93)	0.0016
3	1.81 (1.22 − 2.7)	0.0031	1.69 (1.15 − 2.48)	0.0074
4	3.4 (0.79 − 14.59)	0.081	4.43 (1.05 − 18.78)	0.027
N				
1-3	2.02 (1.54 − 2.66)	2.9e−07	2.07 (1.55 − 2.75)	3.8e−07
1	3.37 (2.07 − 5.49)	2.3e−07	3.01 (1.89 − 4.8)	1.1e−06
2	1.7 (1.08 − 2.66)	0.019	1.61 (1.05 − 2.48)	0.028
3	2.32 (1.33 − 4.07)	0.0025	2.37 (1.32 − 4.26)	0.0028
M				
0	1.91 (1.45 − 2.53)	3.4e−06	1.94 (1.45 − 2.59)	6e−06
1	-	-	-	-
TNM staging				
II	2.11 (1.06 − 4.19)	0.03	1.89 (0.95 − 3.76)	0.067
III	1.7 (1.25 − 2.32)	0.00059	2.09 (1.41 − 3.09)	0.00015
IV	1.56 (1.05 − 2.33)	0.026	1.91 (1.22 − 3)	0.0042
Differentiation				
Moderately-differentiated	2.8 (1.4 − 5.61)	0.0025	2.53 (1.31 − 4.89)	0.0042
Poorly-differentiated	1.62 (0.96 − 2.74)	0.067	2.16 (1.34 − 3.47)	0.0011
Lauren's classification				
Intestinal-type	2.36 (1.72 − 3.25)	5e−08	2.25 (1.53 − 3.32)	2.5e−05
Diffuse-type	1.86 (1.27 − 2.73)	0.0012	1.82 (1.24 − 2.68)	0.0019
Her2 positivity				
−	1.75 (1.37 − 2.24)	6.6e−06	1.95 (1.47 − 2.6)	3.1e−06
+	1.66 (1.23 − 2.24)	9e−04	2.04 (1.47 − 2.84)	1.6e−05
Perforation				
-	1.9 (1.25 − 2.89)	0.0022	2.15 (1.43 − 3.24)	0.00015
Treatment				
Surgery alone	1.81 (1.31 − 2.49)	0.00022	1.7 (1.25 − 2.29)	0.00052
5-FU-based adjuvant	2.18 (1.51 − 3.13)	1.7e−05	2.51 (1.75 − 3.62)	3.1e−07
Other adjuvant	3.01 (1.25 − 7.25)	0.0096	2.9 (1.32 − 6.39)	0.0057

## DISCUSSION

Fascin overexpression was found to promote the proliferation, migration, and invasion of cholangiocarcinoma cells [27]. Darnel et al. [28] found that fascin silencing increased cell adhesive properties, decreased tumor growth, and cell motility and invasiveness, and drastically prevented the formation of lymph node metastases in prostate cancer cells. Further investigation showed that fascin up-regulated NF-κ B activity, uPA, MMP-2 and MMP-9 expression, but down-regulated the expression and nuclear translocation of BRMS1, resulting in a higher ability of breast cancer cells to migrate and invade [29]. In oral squamous cell carcinoma, fascin overexpression led to significant increase in cell migration, cell invasion, and MMP-2 activity with increased levels of phosphorylated Akt, ERK1/2 and JNK1/2 [30]. Liang et al. [31] indicated that fascin promoted the growth and migration of non-small cell lung cancer (NSCLC) cells by activating YAP/TEAD signaling. To investigate the clinicopathological and prognostic significances of fascin expression, we analyzed 11 studies, which met specific inclusion criteria and had moderate to high quality according to their NOS scores.

Chen et al. [32] found that the levels of autoantibody against fascin in the patients with esophageal squamous carcinoma (ESCC) were significantly higher than in control subjects, even for early-stage ESCC. Teng et al. [33] found that the serum fascin level was markedly increased in the NSCLC patients, and positively correlated with aggressive features, including lymphatic and distant metastases. Takikita et al. [34] demonstrated that fascin expression was gradually increased from normal- appearing epithelium to dysplasia to ESCC. Tsai et al. [35] showed that higher fascin immunostaining scores were significantly associated with severe dysplasia of colorectal adenomas and high-grade histopathological differentiation of colorectal adenocarcinomas. Consistent with the data about head and neck squamous cell carcinoma, lung cancer, bladder cancer, cholangiocarcinoma, urothelial carcinoma, laryngeal squamous cell carcinoma, endometrioid carcinoma, hepatocellular carcinoma (HCC), and thyroid cancer [36–44], we found up-regulated fascin expression in gastric cancer, compared with normal mucosa at both mRNA and protein levels. Moreover, fascin expression was positively associated with lymph node metastasis and TNM staging of gastric cancer. In combination with these data, it is suggested that fascin hyperexpression contributes to gastric carcinogenesis and subsequent progression.

Reportedly, fascin overexpression was associated with worse survival of the patients with lung cancer, small intestinal carcinoma, laryngeal squamous cell carcinoma, HCC, oral squamous carcinoma, breast cancer, and ESCC [29, 30, 36, 41, 43, 45, 46]. Fascin expression might be demonstrated to indicate the worse prognosis of cholangiocarcinoma, ovarian cancer, colorectal cancer, extrahepatic bile duct cancer, intrahepatic cholangiocarcinomas, or advanced ovarian serous carcinoma as an independent factor [40, 47–51]. Zhao et al. [52] found that fascin phosphorylation decreased the risk of poor survival in the ESCC patients. Teng et al. [33] demonstrated that the serum fascin level was an independent prognostic factor for M0-stage NSCLC. Our meta-analysis showed that fascin expression was positively linked to the worse prognosis of the patients with gastric cancer. Our bioinformatics data indicated that *FSCN1* mRNA expression was negatively associated with overall and progression-free survival rates of the patient with gastric cancer, even stratified by clinicopathological features. Taken together, the mRNA and protein expression of fascin might be employed as a good and potential marker of the prognosis of the patients with gastric cancer.

In conclusion, fascin expression was upregulated in gastric cancer, and positively correlated with aggressiveness and worse prognosis of the patients with gastric cancer. Several limitations in our meta-analysis included the potential publication bias due from published results being predominantly positive, only Asian patient populations, subjective bias from the extracted survival data from survival curves, and small number of cancer cases in some studies.

## MATERIALS AND METHODS

### Identification of eligible studies and data extraction

We performed a publication search using PubMed, Web of Science, BIOSIS, SciFinder and CNKI updated on March 14, 2017. The following search terms were used: (fascin OR fscn1) AND (gastric OR stomach) AND (cancer OR carcinoma OR adenocarcinoma). Searching was done without restriction on language or publication years. Inclusion criteria for studies: (1) articles to observe the alteration in fascin expression in gastric cancer by immunohistochemistry; (2) papers to compare fascin expression with pathobiological behaviors and prognosis of gastric cancer by immunohistochemistry. Exclusion criteria included: (1) abstract, comment, review and meeting; (2) duplication of the previous publications; (3) Western blot, RT-PCR, cDNA microarray, or transcriptomic sequencing for fascin expression; (4) lack of sufficient information.

### Data extraction

Based on the inclusion criteria, two reviewers (HC Zheng and S Zhao) independently extracted information from all eligible publications. The following information were included in each study: name of first author, year of publication, country, ethnicity, antibody company, numbers of cases and controls, expression alteration, and correlation with survival rate. Regarding survival analysis, we used Engauge Digitizer software to extract data from Kaplan-Meier curves and calculated the Hazard ratios (HR) and their corresponding 95% confidence intervals (CI). Any disagreement was resolved through discussion until the two reviewers reached a consensus.

### Quality score assessment

Two reviewers (HC Zheng and S Zhao) independently assessed the quality of the included studies according to Newcastle Ottawa Scale (NOS) (http://www.ohri.ca/programs/clinical_epidemiology/oxford.htm). The scale consists of three components related to sample selection, comparability and ascertainment of outcome.

### Bioinformatics analysis

The individual gene expression level of *FSCN1* was analyzed using Oncomine (www.oncomine.org), a cancer microarray database and web-based data mining platform for a new discovery from genome-wide expression analyses. We compared the differences in *FSCN1* mRNA level between gastric mucosa and cancer. All data were log-transformed, median centered per array, and standard deviation normalized to one per array. The expression (RNA-seqV2) and clinicopathological data of 392 gastric cancer patients were downloaded from the Cancer Genome Atlas (TCGA) database by TCGA-assembler in R software. We integrated the raw data, analyzed *FSCN1* expression in gastric cancer, and compared it with clinicopathological and prognostic data of the patients with gastric cancer. Additionally, the prognostic significance of *FSCN1* mRNA was also analyzed using Kaplan-Meier plotter (http://kmplot.com).

### Statistics analysis

HWE was evaluated using Chi-square test in control groups of each study. Strength of association between fascin expression and cancer risk was assessed by odds ratios with 95% confidence intervals. Statistical significance of the pooled OR was determined by Z test. If there was no significant heterogeneity, the fixed effect model (Mantel-Haenszel method) would be employed. Otherwise, the random effect model (DerSimonian and Laird method) would be used excluding prognostic analysis. Heterogeneity effect was then quantified by I^2^ test, which was subdivided into low, moderate and high degrees of heterogeneity according to the cut-off values of 25%, 50% and 75% respectively. Publication bias was evaluated by funnel plot and quantified by Begg's test and Egger's test to assess funnel plot asymmetry. Meta-analyses were performed with Revman software 5.3 and data from TCGA database was dealt with SPSS 10.0 software using student t test. *Kaplan-Meier* survival plots were generated and comparisons between survival curves were made with the log-rank statistic. Two-sided p < 0.05 was considered as statistically significant.
